# Treatment strategies for insomnia in Japanese primary care physicians’ practice: A Web-based questionnaire survey

**DOI:** 10.1186/s12875-024-02449-7

**Published:** 2024-06-18

**Authors:** Masahiro Takeshima, Hitoshi Sakurai, Ken Inada, Yumi Aoki, Kenya Ie, Morito Kise, Eriko Yoshida, Kentaro Matsui, Tomohiro Utsumi, Akiyoshi Shimura, Isa Okajima, Nozomu Kotorii, Hidehisa Yamashita, Masahiro Suzuki, Kenichi Kuriyama, Eiji Shimizu, Kazuo Mishima, Koichiro Watanabe, Yoshikazu Takaesu

**Affiliations:** 1 Department of Neuropsychiatry, Akita University Graduate School of Medicine, Akita City, Japan; 2 Department of Neuropsychiatry, Kyorin University Faculty of Medicine, Tokyo, Japan; 3 Department of Psychiatry, Kitasato University School of Medicine, Sagamihara City, Japan; 4 Psychiatric and Mental Health Nursing, Graduate School of Nursing Science, St. Luke’s International University, Tokyo, Japan; 5 Department of General Internal Medicine, St. Marianna University School of Medicine, Kawasaki City, Japan; 6 Department of General Internal Medicine, Kawasaki Municipal Tama Hospital, Kawasaki City, Japan; 7 Centre for Family Medicine Development, Japanese Health and Welfare Co-Operative Federation, Tokyo, Japan; 8 Department of General Internal Medicine, Kawasaki Kyodo Hospital, Kawasaki Health Cooperative Association, Kawasaki City, Japan; 9 Department of Clinical Laboratory, National Center of Neurology and Psychiatry, National Center Hospital, Tokyo, Japan; 10 Department of Sleep-Wake Disorders, National Center of Neurology and Psychiatry, National Institute of Mental Health, Tokyo, Japan; 11 Department of Psychiatry, The Jikei University School of Medicine, Tokyo, Japan; 12 Department of Psychiatry, Tokyo Medical University, Tokyo, Japan; 13 Department of Psychological Counseling, Faculty of Humanities, Tokyo Kasei University, Tokyo, Japan; 14 Kotorii Isahaya Hospital, Isahaya City, Japan; 15 Minnano Sleep and Stress Care Clinic, Hiroshima City, Japan; 16 Department of Psychiatry, Nihon University School of Medicine, Tokyo, Japan; 17 Department of Psychiatry, National Center of Neurology and Psychiatry, National Center Hospital, Tokyo, Japan; 18 Research Center for Child Mental Development, Chiba University, Chiba City, Japan; 19 Department of Cognitive Behavioral Physiology, Chiba University Graduate School of Medicine, Chiba City, Japan; 20 Department of Neuropsychiatry, Graduate School of Medicine, University of the Ryukyus, Nishihara-cho, Japan

**Keywords:** Benzodiazepine, Cognitive behavioral therapy for insomnia, Hypnotic; insomnia, Primary care

## Abstract

**Background:**

It is unclear how primary care physicians manage insomnia after the introduction of novel hypnotics such as orexin receptor antagonists and melatonin receptor agonists. This Web-based questionnaire survey aimed to examine treatment strategies for insomnia in Japanese primary care practice.

**Methods:**

One-hundred-and-seventeen primary care physicians were surveyed on the familiarity of each management option for insomnia on a binary response scale (0 = “unfamiliar”; 1 = “familiar”) and how they managed insomnia using a nine-point Likert scale (1 = “I never prescribe/perform it”; 9 = “I often prescribe/perform it”). Physicians who were unfamiliar with a management option were deemed to have never prescribed or performed it.

**Results:**

Regarding medication, most physicians were familiar with novel hypnotics. Suvorexant was the most used hypnotic, followed by lemborexant and ramelteon. These novel hypnotics averaged 4.8–5.4 points and 4.0–4.7 points for sleep onset and sleep maintenance insomnia, respectively. By contrast, most benzodiazepines were seldom used below two points. Regarding psychotherapy, only approximately 40% of the physicians were familiar with cognitive behavioral therapy for insomnia (CBT-I) and they rarely implemented it, at an average of 1.5–1.6 points. More physicians were familiar with single-component psychotherapies (i.e., relaxation, sleep restriction therapy, and stimulus control) compared to CBT-I, and 48–74% of them implemented it slightly more often, with scores ranging from 2.6 to 3.4 points.

**Conclusion:**

This study suggests that Japanese primary care physicians seldom use CBT-I to treat insomnia. In addition, they use novel sleep medications more frequently than benzodiazepines in terms of pharmacotherapy. The use and availability of CBT-I in Japanese primary care might be facilitated by: educating primary care physicians, implementing brief or digital CBT-I, and/or developing collaborations between primary care physicians and CBT-I specialists.

**Supplementary Information:**

The online version contains supplementary material available at 10.1186/s12875-024-02449-7.

## Background

Insomnia is characterized by symptoms such as difficulty falling and remaining asleep or waking up earlier than desired, as well as distress or daytime dysfunction despite adequate opportunities and suitable environments for sleep [1]. In the Diagnostic and Statistical Manual of Mental Disorders, 5th edition (DSM-5), insomnia is defined as experiencing these symptoms at least three times per week for at least three months [1]. Insomnia is associated with multiple health-related outcomes, including psychiatric disorders such as depression [2–4], anxiety [2, 4], alcohol abuse [2, 5], suicide ideation, [2] suicide attempt, [2] and suicide death [2, 6], as well as physical illnesses such as atrial fibrillation [2, 7], cardiovascular diseases [2, 8], coronary heart diseases, [2, 8] and stroke [2, 8]. Therefore, it is crucial to diagnose it early and provide prompt and appropriate treatment while considering the risks and benefits of each option, as well as the patient’s values, preferences, and circumstances.

Prior studies have reported that 19–36% of primary care patients have chronic insomnia symptoms [9–12]. As chronic insomnia is a common disease and sleep specialists are few, primary care physicians often manage chronic insomnia. [13] The primary treatment for insomnia is psychotherapy, including multi-component cognitive behavioral therapy for insomnia (CBT-I), [14, 15] and pharmacotherapy [16]. Both the American Academy of Sleep Medicine [14] and the European Sleep Research Society guidelines [15] recommend CBT-I as the first-line treatment for chronic insomnia, with pharmacotherapy recommended only when CBT-I proves to be ineffective or is not accessible. However, primary care physicians rarely use CBT-I to treat chronic insomnia [17, 18] because it is burdensome for them, as it is typically administered over four to eight sessions that last 30–120 min [14, 19]; moreover, primary care physicians lack knowledge of CBT-I [18]. Thus, education and policy changes regarding training for and delivery of CBT-I are needed.

Benzodiazepine receptor agonists (BzRA), which consist of benzodiazepines and Z-drugs, are effective treatments for chronic insomnia in the short term [20]. However, long-term prescription of BzRA is not recommended [15, 16] because its long-term efficacy for chronic insomnia has not been established [20], and numerous studies have shown that long-term use of BzRA increases the risk of falls [21], fractures [22], and cognitive dysfunction [23]. Despite physicians understanding these risks [18, 24], BzRA continues to be prescribed for long-term use worldwide [25–27]. An Australian study reported that, in general practice, long-term prescriptions of BzRA (defined as receiving at least three prescriptions of BzRA within 180 days, with the second prescription prescribed after 28 days of the first) ranged from 4.4% to 5.8% between 2011 and 2018 [27]. As primary care physicians often treat insomnia with pharmacotherapy [18], clarifying how primary care physicians discontinue BzRA may help reduce the long-term prescribing of BzRA. However, primary care physicians' BzRA discontinuation strategies have not been studied to date.

In recent years, novel sleep medications, such as orexin receptor antagonists (ORAs) and melatonin receptor agonists (MRAs), have been introduced. These medications are considered safe because they do not have side effects, such as dependence, as with BzRA [28–30]. ORAs were evaluated as both effective and safe sleep medication in two recent network meta-analyses [20, 31]. However, the position of these novel sleep medications in primary care remains unclear.

A previous study based on questionnaires and qualitative interviews published in 2014 reported that general practitioners (GPs) in the United Kingdom rarely use psychotherapies such as CBT-I for insomnia and often reluctantly prescribe benzodiazepines and Z-drugs under pressure from patients [18]. However, the study did not examine primary care physicians' prescribing practices for novel hypnotics, nor did it examine the extent to which primary care physicians use individual psychotherapy to treat insomnia or how they discontinue BzRA [18]. Furthermore, in the nearly 10 years since the study was conducted [18], insomnia guidelines have been published and novel sleep medications have been developed, but how primary care physicians have managed insomnia in recent years has not been studied. To treat insomnia appropriately and prevent long-term BzRA prescription in primary care settings, it is important to understand the actual management of insomnia by primary care physicians and develop countermeasures to address the identified problems.

Therefore, this survey was conducted to investigate the actual management of insomnia (pharmacotherapy and psychotherapy as well as BzRA discontinuation strategies) by Japanese primary care physicians.

## Methods

### Participants and procedure

This cross-sectional questionnaire survey aimed to examine the actual management of insomnia by Japanese primary care physicians. Primary care physicians on the mailing list of the Japan Primary Care Association (JPCA) were invited to participate in the questionnaire survey via email from July 15, 2022, to August 26, 2022. Japan has universal health insurance, and its citizens have free access to medical care. The role of primary care physicians in Japan is to provide comprehensive medical care that is close to the people, render consultation services on any matter, as well as take responsibility for community health care. The primary care physicians who agreed to participate in this study were asked how they managed insomnia without psychiatric comorbidity according to DSM-5. This study is not limited to insomnia with/without physical comorbidity.

### Questionnaire

A task force of three primary care physicians and 11 specialists for insomnia was formed to identify clinical questions regarding the management of insomnia disorder without psychiatric comorbidity based on DSM-5 (1) in primary care settings. After a thorough discussion, the task force established the following ten clinical question items that are common in practice: (1) pharmacological strategies for sleep onset insomnia, (2) non-pharmacological strategies for sleep onset insomnia, (3) pharmacological strategies for sleep maintenance insomnia, (4) non-pharmacological strategies for sleep maintenance insomnia, (5) pharmacological strategies when insomnia symptoms do not improve with BzRA, (6) non-pharmacological strategies when insomnia symptoms do not improve with BzRA, (7) preferred timing to start BzRA reduction after insomnia symptoms improve, (8) methods used to discontinue BzRA, (9) medications when discontinuing BzRA by switching to other drugs, and (10) which patients with insomnia are acceptable to continue BzRA. Multiple management choices were presented for each clinical question. Participants were first asked if they were familiar with each choice. If the participant was familiar with the choice, they responded to each management option on a nine-point Likert scale (1 = “I do not prescribe or perform it at all” to 9 = “I often prescribe or perform it”). If a participant was not familiar with the option, they were considered to not be prescribing or performing it at all. Details of the questionnaire used in this study are presented in Supplementary Table 1. The following pharmacological treatments for insomnia were determined through task force discussions (ramelteon, suvorexant, lemborexant, eszopiclone, zopiclone, zolpidem, etizolam, triazolam, flunitrazepam, brotizolam, nitrazepam, trazodone, quetiapine, and traditional Chinese medicine (TCM)). Brotizolam is a short-acting benzodiazepine with a half-life of 7 h. All sleep medications that were classified as Z-drugs, MRAs, and ORAs, as well as the five most commonly prescribed benzodiazepines based on unpublished data from a secondary analysis of our previous study [26], were selected. In addition, trazodone, quetiapine, and TCM, which are not classified as sleep medications but may be prescribed for insomnia, were also selected. As barbiturates are rarely used for insomnia in Japan [26] and are not recommended by guidelines [15, 16], they were not included in the survey. Participants were also asked to specify their age and sex, as well as the academic societies other than JPCA to which they belong. Participants were not asked to provide their names. The survey took approximately 15 min to complete. The physicians who participated in this survey were unpaid volunteers.

### Statistical analysis

All statistical analyses were performed using SPSS Statistics 28.0 (IBM Corp. Armonk, NY, USA). The percentage of cases wherein participants were unfamiliar with various treatment options was examined. In addition, the percentage of “unfamiliar” responses was calculated for all pharmacologic treatments, all non-pharmacologic treatments, all non-pharmacologic treatments except for CBT-I, and all pharmacologic and non-pharmacologic treatments for sleep onset or sleep maintenance insomnia. Mean, standard deviation, and 95% confidence interval (CI) were calculated for each management choice.

### Ethics

This study was approved by the Ethics Committee of St. Luke’s International University (No. 2021–604). Online informed consent was obtained prior to questionnaire access for all participants.

## Results

### Participant characteristics

In this survey, 117 primary care physicians completed the questionnaire. The response rate was 2.2% (117/5306). The median age of the participants was 47 years (interquartile range: 39–55 years). The proportions of male and female participants were 76.1% and 18.8%, respectively. Of the participants, 26.5% were members of societies other than JPCA, 2.6% were members of societies related to psychiatry, and 0.9% were members of the Japanese Society of Sleep Research (Table 1).

**Table 1:** Characteristics of the participants

	Participants (*N* = 117)
***Age***	47 (39–55)
***Sex***	
Male	76.1% (89)
Female	18.8% (22)
Non-response	5.1% (6)
***Academic Society***	
Japanese society of internal medicine	17.1% (20)
Japanese association for home care medication	5.1% (6)
Japanese society of hospital general medicine	4.3% (5)
Japan geriatrics society	3.4% (4)
Japan diabetes society	1.7% (2)
Japan society for oriental medicine	1.7% (2)
Japanese society of obstetrics and gynecology	1.7% (2)
Japanese circulation society	1.7% (2)
Japanese society of sleep research	1.7% (2)

Values are presented as median (interquartile range) or percentage (number)

Table 1 lists the academic societies that have two or more participants.

### Pharmacological strategies for insomnia

Regarding pharmacotherapy, for both sleep onset and maintenance insomnia, ORAs and MRAs were familiar to approximately ≥ 90% of primary care physicians, Z-drugs were familiar to > 80%, while benzodiazepines (other than brotizolam) were familiar to < 80%. All primary care physicians were familiar with either pharmacologic treatment options for sleep onset and sleep maintenance insomnia. Regarding sleep onset insomnia, suvorexant was used most frequently, with a score of 5.4 ± 2.5 (95%CI: 5.0–5.9); followed by lemborexant [5.2 ± 3.0 (95%CI: 4.6–5.7)]; ramelteon [4.8 ± 2.4 (95%CI: 4.4–5.3)]; zolpidem [4.1 ± 2.6 (95%CI: 3.6–4.5)]; eszopiclone [4.0 ± 2.6 (95%CI: 3.5–4.5)]; traditional Chinese medicine [3.3 ± 2.6 (95%CI: 2.9–3.8)]; trazodone [3.2 ± 2.5 (95%CI: 2.8–3.7)]; and quetiapine [2.6 ± 2.2 (95%CI: 2.2–3.0)]. In general, benzodiazepines were rarely used with an average 1-point range, except for brotizolam [2.9 ± 2.2 (95%CI: 2.5–3.3)]. Regarding sleep maintenance insomnia, suvorexant was used most frequently, with a score of 4.7 ± 2.7 (95%CI: 4.2–5.2); followed by lemborexant [4.6 ± 2.9 (95%CI: 4.1–5.2)]; ramelteon [4.0 ± 2.5 (95%CI: 3.5–4.4)]; trazodone [3.3 ± 2.5 (95%CI: 2.8–3.8)]; traditional Chinese medicine [3.1 ± 2.6 (95%CI: 2.6–3.6)]; eszopiclone [2.9 ± 2.4 (95%CI: 2.5–3.4)]; brotizolam [2.7 ± 2.2 (95%CI: 2.3–3.1)]; zolpidem [2.5 ± 1.9 (95%CI: 2.1–2.8)]; quetiapine [2.2 ± 2.0 (95%CI: 1.8–2.6)]; and zopiclone [2.1 ± 0.7 (95%CI: 1.8–2.4)]. As with sleep onset insomnia, benzodiazepines other than brotizolam were rarely used for sleep maintenance insomnia (Table 2, Figures S1–2).

**Table 2:** Pharmacological and non-pharmacological treatments for sleep onset and maintenance insomnia

	Sleep onset insomnia			Sleep maintenance insomnia		
	Familiarity	Mean (SD)	95%CI	Familiarity	Mean (SD)	95%CI
***Pharmacological treatments***						
Suvorexant	97.4% (114)	5.4 (2.5)	5.0–5.9	95.7% (112)	4.7 (2.7)	4.2–5.2
Lemborexant	91.5% (107)	5.2 (3.0)	4.6–5.7	89.7% (105)	4.6 (2.9)	4.1–5.2
Ramelteon	97.4% (114)	4.8 (2.4)	4.4–5.3	91.5% (107)	4.0 (2.5)	3.5–4.4
Zolpidem	92.3% (108)	4.1 (2.6)	3.6–4.5	81.2% (95)	2.5 (1.9)	2.1–2.8
Eszopiclone	93.2% (109)	4.0 (2.6)	3.5–4.5	87.2% (102)	2.9 (2.4)	2.5–3.4
TCM	79.5% (93)	3.3 (2.6)	2.9–3.8	79.5% (93)	3.1 (2.6)	2.6–3.6
Trazodone	83.8% (98)	3.2 (2.5)	2.8–3.7	81.2% (95)	3.3 (2.5)	2.8–3.8
Brotizolam	83.8% (98)	2.9 (2.2)	2.5–3.3	80.3% (94)	2.7 (2.2)	2.3–3.1
Zopiclone	90.6% (106)	2.7 (2.1)	2.3–3.1	80.3% (94)	2.1 (1.7)	1.8–2.4
Quetiapine	79.5% (93)	2.6 (2.2)	2.2–3.0	74.4% (87)	2.2 (2.0)	1.8–2.6
Etizolam	76.1% (89)	1.9 (1.7)	1.6–2.3	69.2% (81)	1.7 (1.4)	1.4–2.0
Flunitrazepam	67.5% (79)	1.5 (1.2)	1.3–1.7	71.8% (84)	1.9 (1.6)	1.6–2.2
Nitrazepam	61.5% (72)	1.5 (1.1)	1.3–1.7	65.8% (77)	1.7 (1.5)	1.4–1.9
Triazolam	64.1% (75)	1.4 (0.9)	1.2–1.5	62.4% (73)	1.2 (0.8)	1.1–1.4
Any pharmacologic treatments	100% (117)	NA	NA	100% (117)	NA	NA
***Non-pharmacological treatments***						
Sleep hygiene education	94.0% (110)	6.4 (2.7)	5.9–6.9	88.9% (104)	5.4 (2.9)	4.9–6.0
Relaxation therapy	74.0% (87)	3.4 (2.7)	2.9–4.0	70.1% (82)	3.4 (2.7)	2.9–3.9
Stimulus control	59.0% (69)	2.9 (2.7)	2.5–3.4	48.7% (57)	2.6 (2.6)	2.1–3.1
Sleep restriction therapy	53.0% (62)	2.9 (2.5)	2.4–3.4	58.1% (68)	2.9 (2.6)	2.4–3.4
Multi-component CBT-I	39.3% (46)	1.5 (1.3)	1.3–1.7	38.5% (45)	1.6 (1.5)	1.3–1.8
Any non-pharmacologic treatments	94.0% (110)	NA	NA	94.0% (110)	NA	NA
Any non-pharmacologic treatments other than sleep hygiene education	82.1% (96)	NA	NA	82.1% (96)	NA	NA

Values are presented as percentage (number) or mean (standard deviation)

*Abbreviation: CBT-I* cognitive behavioral therapy for insomnia, *CI* confidence interval, *NA* not applicable, *SD* standard deviation, *TCM* traditional Chinese medicine

### Non-pharmacological strategies for insomnia

Regarding psychosocial therapy, for both sleep onset and maintenance insomnia, the primary care physicians were familiar with sleep hygiene education, relaxation, stimulus control, sleep restriction therapy, and CBT-I, in that order. Furthermore, 94.0% of the physicians were familiar with any non-pharmacologic treatment option, while 82.1% were familiar with any non-pharmacologic treatment option other than sleep hygiene education. For sleep onset insomnia, sleep hygiene education was practiced most often, with a score of 6.4 ± 2.7 (95%CI: 5.9–6.9), followed by relaxation, stimulus control, sleep restriction therapy, and CBT-I [3.4 ± 2.7 (95%CI: 2.9–4.0), 2.9 ± 2.7 (95%CI: 2.5–3.4), 2.9 ± 2.5 (95%CI: 2.4–3.4), and 1.5 ± 1.3 (95%CI: 1.3–1.7), respectively]. For sleep maintenance insomnia, sleep hygiene education was practiced the most, with a score of 5.4 ± 2.9 (95%CI: 4.9–6.0), followed by relaxation, sleep restriction therapy, and stimulus control [3.4 ± 2.7 (95%CI: 2.9–3.9), 2.9 ± 2.6 (95%CI: 2.4–3.4), and 2.6 ± 2.6 (95%CI: 2.1–3.1), respectively]. CBT-I was seldom practiced, with a score of 1.6 ± 1.5 (95%CI: 1.3–1.8) (Table 2, Figures S3–4).

### Strategies when insomnia does not improve with BzRA

For pharmacotherapy, primary care physicians implemented a combination therapy with novel sleep medications the most frequently when insomnia symptoms did not improve with BzRA; the scores were as follows: combination therapy with suvorexant: 4.6 ± 2.5 (95%CI: 4.1–5.1); with lemborexant: 4.4 ± 2.8 (95%CI: 3.9–4.9); and with ramelteon: 4.2 ± 2.6 (95%CI: 3.8–4.7). Primary care physicians were least likely to switch to quetiapine [2.2 ± 1.8 (95%CI: 1.9–2.5)], concomitant use of quetiapine [2.3 ± 1.9 (95%CI: 2.0–2.7)], or concomitant use of other BzRA [2.3 ± 2.0 95%CI: 2.0–2.7)] in patients whose insomnia symptoms did not improve with BzRA. Regarding non-pharmacotherapy strategies, the scores were as follows: sleep hygiene education [5.7 ± 3.0 (95%CI: 5.1–6.2)], differential diagnosis of comorbid psychiatric disorders [5.6 ± 2.7 (95%CI: 5.1–6.1)], differentiation of other sleep disorders [5.6 ± 2.7 (95%CI: 5.1–6.1)], referral to a specialist hospital [3.6 ± 2.6 (95%CI: 3.2–4.1)], relaxation [3.4 ± 2.8 (95%CI: 2.9–3.9)], stimulus control [2.9 ± 2.7 (95%CI: 2.4–3.4)], sleep restriction therapy [2.8 ± 2.6 (95%CI: 2.3–3.3)], and CBT-I [1.6 ± 1.6 (95%CI: 1.3–1.9)] (Table 3).

**Table 3:** Pharmacological and non-pharmacological treatments when insomnia symptoms do not improve with benzodiazepine receptor agonists

	Familiarity	Mean (SD)	95%CI
***Pharmacological treatments***			
Combination of suvorexant	94.0% (110)	4.6 (2.5)	4.1–5.1
Combination of lemborexant	87.2% (102)	4.4 (2.8)	3.9–4.9
Combination of ramelteon	90.6% (106)	4.2 (2.6)	3.8–4.7
Switching to suvorexant	94.9% (111)	4.0 (2.6)	3.5–4.5
Switching to lemborexant	87.2% (102)	4.0 (2.8)	3.4–4.5
Switching to other BzRA	88.0% (103)	3.5 (2.4)	3.1–4.0
Combinations with trazodone	83.8% (98)	3.5 (2.6)	3.0–4.0
Switching to ramelteon	89.7% (105)	3.3 (2.3)	2.9–3.7
Switching to trazodone	84.6% (99)	3.2 (2.6)	2.9–3.7
Increasing BzRA dosage	85.5% (100)	3.1 (2.3)	2.7–3.5
Combinations with quetiapine	78.6% (92)	2.3 (1.9)	2.0–2.7
Combinations with other BzRA	79.5% (93)	2.3 (2.0)	2.0–2.7
Switching to quetiapine	81.2% (95)	2.2 (1.8)	1.9–2.5
***Non-pharmacological treatments***			
Sleep hygiene education	88.9% (104)	5.7 (3.0)	5.1–6.2
Differential diagnosis of comorbid psychiatric disorders	95.7% (112)	5.6 (2.7)	5.1–6.1
Differentiating other sleep disorders	96.6% (113)	5.6 (2.7)	5.1–6.1
Referring to a specialist hospital	89.7% (105)	3.6 (2.6)	3.2–4.1
Relaxation therapy	70.9% (83)	3.4 (2.8)	2.9–3.9
Stimulus control	53.0% (62)	2.9 (2.7)	2.4–3.4
Sleep restriction therapy	57.3% (67)	2.8 (2.6)	2.3–3.3
Multi-component CBT-I	36.8% (43)	1.6 (1.6)	1.3–1.9

Values are presented as percentage (number) or mean (standard deviation)

*Abbreviation: BzRA* benzodiazepine receptor agonists, *CBT-I* cognitive behavioral therapy for insomnia, *CI* confidence interval, *SD* standard deviation

### Discontinuation of BzRA

Regarding the preferred time to start BzRA reduction after insomnia improvement, 1–3 months received the highest score [5.1 ± 2.3 (95%CI: 4.7–5.6)], followed by 3–6 months [4.6 ± 2.3 (95%CI: 4.2–5.1)], 6–12 months [3.9 ± 2.2 (95%CI: 3.5–4.3)], 12 + months [3.7 ± 2.4 (95%CI: 3.3–4.2)], and immediately after improvement [3.2 ± 2.3 (95%CI: 2.8–3.6)] (Table 4). For methods used to discontinue BzRA, primary care physicians used controlled gradual tapering the most [6.8 ± 2.4 (95%CI: 6.3–7.2)], followed by switching to another medication [5.6 ± 2.5 (95%CI: 5.2–6.1)], sleep hygiene education [5.1 ± 2.9 (95%CI: 4.6–5.7)], switching to “as needed” medication [5.1 ± 2.6 (95%CI: 4.7–5.6)], and self-tapering [4.6 ± 2.5 (95%CI: 4.2–5.1)]. Primary care physicians provided occasional psychotherapy other than sleep hygiene education in BzRA discontinuation, and CBT-I was rarely implemented [1.5 ± 1.4 (95%CI: 1.2 to 1.7)] (Table 4). For pharmacotherapy when discontinuing BzRA by switching to other drugs, primary care physicians used suvorexant [5.3 ± 2.7 (95%CI: 4.8–5.8)], lemborexant [5.0 ± 2.8 (95%CI: 4.5–5.5)], ramelteon [4.6 ± 2.6 (95%CI: 4.1–5.1)], trazodone [3.5 ± 2.7 (95%CI: 3.0–4.0)], traditional Chinese medicine [3.1 ± 2.6 (95%CI: 2.6–3.6)], and quetiapine [2.3 ± 2.0 (95%CI: 1.9–2.7)] (Table 4). Regarding patients with insomnia who continued BzRA, patients’ preference to continue BzRA was the biggest factor for BzRA continuation [6.4 ± 2.1 (95%CI: 6.0–6.7)], followed by anticipation of physical or mental deterioration upon discontinuing BzRA [6.3 ± 2.2 (95%CI: 5.9–6.7)], history of exacerbation of insomnia symptoms after discontinuing BzRA [6.2 ± 2.1 (95%CI: 5.8–6.6)], BzRA being used as monotherapy or used at low doses [5.6 ± 2.4 (95%CI: 5.1–6.0)], unstable physical or mental states or low quality of life [5.5 ± 2.2 (95%CI: 5.1–5.9)], and lack of awareness of side effects [4.5 ± 2.4 (95%CI: 4.1–4.9) (Table 4).

**Table 4:** Discontinuation strategies of benzodiazepine receptor agonists

	Familiarity	Mean (SD)	95%CI
***Preferred timing to start BzRA reduction***			
After 1–3 month(s)	NA	5.1 (2.3)	4.7–5.6
After 3–6 months	NA	4.6 (2.3)	4.2–5.1
After 6–12 months	NA	3.9 (2.2)	3.5–4.3
After 12 + months	NA	3.7 (2.4)	3.3–4.2
Immediately after improvement	NA	3.2 (2.3)	2.8–3.6
***Methods used to discontinue BzRA***			
Controlled gradual tapering	98.3% (115)	6.8 (2.4)	6.3–7.2
Switching to another medication	94.9% (111)	5.6 (2.5)	5.2–6.1
Sleep hygiene education	89.7% (105)	5.1 (2.9)	4.6–5.7
Switching to “as needed” medication	95.7% (112)	5.1 (2.6)	4.7–5.6
Self-tapering	94.0% (110)	4.6 (2.5)	4.2–5.1
Relaxation therapy	70.9% (83)	3.1 (2.7)	2.6–3.6
Stimulus control	59.0% (69)	2.7 (2.6)	2.2–3.2
Sleep restriction therapy	59.8% (70)	2.6 (2.5)	2.2–3.1
Multi-component CBT-I	40.2% (47)	1.5 (1.4)	1.2–1.7
***Medications used when discontinuing BzRA by switching to other drugs***			
Suvorexant	93.2% (109)	5.3 (2.7)	4.8–5.8
Lemborexant	88.9% (104)	5.0 (2.8)	4.5–5.5
Ramelteon	92.3% (108)	4.6 (2.6)	4.1–5.1
Trazodone	85.5% (100)	3.5 (2.7)	3.0–4.0
TCM	77.8% (91)	3.1 (2.6)	2.6–3.6
Quetiapine	81.2% (95)	2.3 (2.0)	1.9–2.7
***Which patients with insomnia are acceptable to continue BzRA***			
Patient’s preference to continue BzRA	NA	6.4 (2.1)	6.0–6.7
Anticipation of physical and mental deterioration	NA	6.3 (2.2)	5.9–6.7
History of exacerbation of insomnia symptoms	NA	6.2 (2.1)	5.8–6.6
BzRA being used as monotherapy or at low doses	NA	5.6 (2.4)	5.1–6.0
Unstable physical or mental states or low quality of life	NA	5.5 (2.2)	5.1–5.9
Lack of awareness of side effects	NA	4.5 (2.4)	4.1–4.9

Values are presented as percentage (number) or mean (standard deviation)

*Abbreviation: BzRA* benzodiazepine receptor agonists, *CBT-I* cognitive behavioral therapy for insomnia, *CI* confidence interval, *NA* not applicable, *SD* standard deviation, *TCM* traditional Chinese medicine

## Discussion

This was the first study to examine in detail the management of insomnia by primary care physicians after the introduction of novel sleep medications. Consistent with the 2014 questionnaire survey of UK GPs [18], Japanese primary care physicians did not widely use psychosocial therapies, except for sleep hygiene education, for insomnia. In contrast to the same survey [18], novel sleep medications, rather than BzRAs, were commonly used.

The study showed that primary care physicians generally used ORAs, MRAs, Z-drugs, and benzodiazepines, in that order, to treat sleep onset and sleep maintenance insomnia. Whether the participants’ prescribing trends reflect the broader medical situation remains unclear, as there are no studies on the actual prescribing of sleep medications in Japan for 2022, when this survey was conducted. A pharmacoepidemiologic study using a nationwide Japanese claims database reported that between 2010 and 2019, benzodiazepine prescriptions decreased from 54.8% to 30.5%, MRAs increased slightly from 3.2% to 6.3%, ORAs increased significantly from 0% to 20.2%, and Z-drugs remained stable at approximately 40% [32]. Considering prescribing trends in the previous study [32], Japanese primary care physicians seem to prefer novel sleep medications. The results of this study on ORAs align with those of a systematic review and network meta-analysis (NMA) published in 2023 by Yue et al [31]. In the NMA study, ORAs ranked best for both sleep onset and sleep maintenance insomnia, and second-best for tolerability among all classes of sleep medications based on the surface under the cumulative ranking curve values [31]. Concerning MRAs and Z-drugs, this study showed that primary care physicians preferred MRAs over Z-drugs for treating insomnia [31]. These results are interesting because a prior NMA’s results were more favorable to Z-drugs than to MRAs [20, 31]. Yue et al. concluded that Z-drugs are effective for both sleep onset and sleep maintenance insomnia, [31], and an NMA by Crescenzo et al. concluded that eszopiclone, which is classified as a Z-drug, has a good profile along with lemborexant [20]. In addition, Yue et al. showed that ramelteon is inferior to many sleep medications for the treatment of sleep maintenance insomnia [31], and Crescenzo et al. concluded that ramelteon has no material benefit in the treatment of insomnia [20]. One possible reason for the divergence between evidence and primary care physicians’ prescription-related behavior could be the high safety profile of ramelteon [31]. Interestingly, in our previous survey with 762 Japanese physicians, we found that physicians who frequently prescribed ramelteon were more concerned about safety when choosing sleep medication to treat insomnia than physicians who did not prescribe ramelteon frequently [24]. In pharmacotherapy for insomnia, primary care physicians may be more safety conscious because they often treat insomnia with physical comorbidities [13, 33]. As this study did not examine whether the presence or absence of physical comorbidities changes the primary care physician's strategy for treating insomnia, additional research is needed to further elucidate primary physicians’ prescribing behavior in this regard.

Despite the fact that CBT-I is the recommended first-line treatment for insomnia, this study found that Japanese primary care physicians not only rarely used CBT-I to treat insomnia but were also unfamiliar with it. The results of this study are consistent with a previous study of GPs in the United Kingdom [18]. The reasons for such lack of familiarity are unclear despite the recommendation of CBT-I as the first-line treatment for insomnia [14, 15]. This may be because few medical institutions in Japan offer CBT-I and it is not covered by insurance [34]. Notably, more primary care physicians were familiar with and implemented relaxation therapy, sleep restriction therapy, and stimulus control than with CBT-I. These single-component psychotherapies have been suggested to be effective on their own [14] and may be more acceptable to primary care physicians because they may be easier to administer and learn than CBT-I. Nevertheless, CBT-I is recommended as the first-line treatment for insomnia based on existing evidence [14, 15]. To deliver CBT-I to patients in Japanese primary care settings, primary care physicians must first understand CBT-I, its efficacy, and its safety [35]. Then, training primary care physicians to perform CBT-I and establishing links between primary care physicians and CBT-I specialists [13] would make it easier for patients in primary care settings to receive CBT-I. Furthermore, since CBT-I is more burdensome for primary care physicians who often treat diseases other than insomnia [13], the development and social implementation of brief or digital CBT-I is also desirable [17, 36]. Regarding sleep hygiene education, Japanese primary care physicians frequently provided sleep hygiene education, similar to the results from the survey of GPs in the United Kingdom [18]. A clinical practice guideline of the American Academy of Sleep Medicine recommends not using sleep hygiene education as a single-component therapy for the treatment of chronic insomnia disorder [14]. This study examined the frequency with which Japanese primary care physicians offer each treatment option for insomnia, but it did not examine whether each treatment option is used alone or in combination. Future studies are needed to determine whether primary care physicians are treating insomnia in accordance with guidelines.

Primary care physicians often prescribed ORAs in combination or as substitutions for drug therapy when BzRA did not improve insomnia. In addition, sleep hygiene education was often used as psychotherapy, but sleep restriction therapy and stimulus control were not often used, and CBT-I, which encompassed these therapies, was seldom utilized. This indicates that there was no difference between the primary care physicians’ strategies for treating sleep onset or maintenance insomnia and their strategies for addressing insomnia that did not improve with BzRA use. When insomnia did not improve with BzRA, primary care physicians identified other sleep disorders and psychiatric comorbidities but were less likely to refer patients to facilities specializing in sleep disorders. In the Japanese expert consensus on the treatment of insomnia, if insomnia did not improve with BzRA use, then identifications of sleep disorders and psychiatric comorbidities were categorized as first-line recommendations, while referral to a facility specializing in sleep disorders was categorized as a second-line recommendation [37]. Why primary care physicians made few referrals to sleep specialty institutions in this study remains unclear, but this may be because primary care physicians are unaware of specialized treatments such as CBT-I [13], and there are few sleep specialty institutions [38]. Thus, the ability of patients to access CBT-I within primary care settings is unlikely. Therefore, when insomnia does not improve with sleeping medications, it is better to refer the patient to a specialized medical facility. Future research is warranted to identify barriers to referral by primary care physicians to sleep specialty providers.

### Limitations

This study had several limitations. First, not all Japanese primary care physicians are affiliated with JPCA, and the response rate for this survey was low, reaching only 2.2%; thus, the limited number of participants prevents generalization to all primary care physicians. Second, the findings relied on subjective survey responses. In this study, participants responded to the frequency of prescribing or performing each management option on a nine-point Likert scale, but this was not strictly defined. Therefore, there could be discrepancies in responses among participants. Third, the questionnaire presents terminology without any explanations or examples. In an Australian qualitative study, some GPs offered variations of sleep restriction therapy, but the study did not identify sleep restriction therapy as a component of CBT-I [13]. Thus, the interpretation of the meaning of these terms could affect the results. Fourth, because this study did not strictly define the psychotherapies, their methods may have differed among the participants. Physicians who performed certain psychotherapies using simple methods may have done so more often than those who did not. Fifth, it is not possible to generalize the results of this study to primary care physicians worldwide because all participants practiced in Japan. In conclusion, this study suggests that Japanese primary care physicians seldom used CBT-I to treat insomnia. In addition, they frequently used novel sleep medications more than benzodiazepines in terms of pharmacotherapy. Making CBT-I available in Japan through primary care could be facilitated by: educating primary care physicians, implementing brief or digital CBT-I, and/or establishing collaborations between primary care physicians and CBT-I specialists.

### Supplementary Information

Additional file 1: Figure S1. Pharmacological strategies for sleep onset insomnia. Familiarity indicated the number and percentage of those who knew each pharmacological treatment for sleep onset insomnia. If the participant was familiar with the treatment, they responded to each management option on a nine-point Likert scale (1 = “I do not prescribe it at all”; 9 = “I often prescribe it”). If a participant was not familiar with the option, they were considered to have not prescribed the option at all. Abbreviations: CI, confidence interval; SD, standard deviation; TCM, traditional Chinese medicine. Figure S2. Pharmacological strategies for sleep maintenance insomnia. Familiarity indicated the number and percentage of those who knew each pharmacological treatment for sleep maintenance insomnia. If the participant was familiar with the treatment, they responded to each management option on a nine-point Likert scale (1 = “I do not prescribe it at all”; 9 = “I often prescribe it”). If a participant was not familiar with the option, they were considered to have not prescribed the option at all. Abbreviations: CI, confidence interval; SD, standard deviation; TCM, traditional Chinese medicine. Figure S3. Non-pharmacological strategies for sleep onset insomnia. Familiarity indicated the number and percentage of those who knew each non-pharmacological treatment for sleep onset insomnia. If the participant was familiar with the treatment, they responded to each management option on a nine-point Likert scale (1 = “I do not perform it at all”; 9 = “I often perform it”). If a participant was not familiar with the option, they were considered to have not performed the option at all. Abbreviations: CBT-I, cognitive behavioral therapy for insomnia; CI, confidence interval; SD, standard deviation. Figure S4. Non-pharmacological strategies for sleep maintenance insomnia. Familiarity indicated the number and percentage of those who knew each non-pharmacological treatment for sleep maintenance insomnia. If the participant was familiar with the treatment, they responded to each management option on a nine-point Likert scale (1 = “I do not perform it at all”; 9 = “I often perform it”). If a participant was not familiar with the option, they were considered to have not performed the option at all. Abbreviations: CBT-I, cognitive behavioral therapy for insomnia; CI, confidence interval; SD, standard deviation. Table S1. Details of the questionnaire

## Data Availability

The datasets supporting the conclusions of this article are available from the corresponding author upon reasonable request.

## Abbreviations

BzRA: Benzodiazepine receptor agonists
CBT-I: Cognitive behavioral therapy for insomnia
CI: Confidence interval
GP: General practitioners
JPCA: Japan Primary Care Association
MRA: Melatonin receptor agonist
NMA: Network meta-analysis
ORA: Orexin receptor antagonist
SD: Standard deviation
TCM: Traditional Chinese medicine
