# Supplementary material for: Treatment strategies for insomnia in Japanese primary care physicians’ practice: A Web-based questionnaire survey
Source: BMC Prim Care. 2024 Jun 18;25:219. doi: 10.1186/s12875-024-02449-7 (PMC11184713; doi:10.1186/s12875-024-02449-7)
Supplement: Supplementary file 1 — Additional file 1: Figure S1. Pharmacological strategies for sleep onset insomnia. Familiarity indicated the number and percentage of those who knew each pharmacological treatment for sleep onset insomnia. If the participant was familiar with the treatment, they responded to each management option on a nine-point Likert scale (1 = “I do not prescribe it at all”; 9 = “I often prescribe it”). If a participant was not familiar with the option, they were considered to have not prescribed the option at all. Abbreviations: CI, confidence interval; SD, standard deviation; TCM, traditional Chinese medicine. Figure S2. Pharmacological strategies for sleep maintenance insomnia. Familiarity indicated the number and percentage of those who knew each pharmacological treatment for sleep maintenance insomnia. If the participant was familiar with the treatment, they responded to each management option on a nine-point Likert scale (1 = “I do not prescribe it at all”; 9 = “I often prescribe it”). If a participant was not familiar with the option, they were considered to have not prescribed the option at all. Abbreviations: CI, confidence interval; SD, standard deviation; TCM, traditional Chinese medicine. Figure S3. Non-pharmacological strategies for sleep onset insomnia. Familiarity indicated the number and percentage of those who knew each non-pharmacological treatment for sleep onset insomnia. If the participant was familiar with the treatment, they responded to each management option on a nine-point Likert scale (1 = “I do not perform it at all”; 9 = “I often perform it”). If a participant was not familiar with the option, they were considered to have not performed the option at all. Abbreviations: CBT-I, cognitive behavioral therapy for insomnia; CI, confidence interval; SD, standard deviation. Figure S4. Non-pharmacological strategies for sleep maintenance insomnia. Familiarity indicated the number and percentage of those who knew each non-pharmacological treatment for sleep ma [file 12875_2024_2449_MOESM1_ESM.zip › Table S1.docx]

**Supplementary Table 1. Details of the questionnaire**

In this questionnaire, we will inquire about your treatment choices for insomnia disorder without psychiatric comorbidity based on DSM-5 with the following two questions:

(A) Please indicate your familiarity with each option for the given situation (Q1, Q2, Q3, Q4, Q5, Q6, Q8, and Q9).

0 　1

Unfamiliar Familiar

(B) If familiar, please rate how often you have used each option for the given situation (Q1–10).

1 2 3 4 5 6 7 8 9

←Never 　　　　　　Frequently→

Q1) To what extent do you prescribe the following pharmacological treatments for sleep onset insomnia?

- Ramelteon
- Suvorexant
- Lemborexant
- Eszopiclone
- Zopiclone
- Zolpidem
- Etizolam
- Triazolam
- Flunitrazepam
- Brotizolam
- Nitrazepam
- Trazodone
- Quetiapine
- Traditional Chinese medicine

Q2) To what extent do you implement the following non-pharmacological treatments for sleep onset insomnia?

- Sleep hygiene education
- Relaxation therapy
- Stimulus control
- Sleep restriction therapy
- Multi-component cognitive behavioral therapy for insomnia

Q3) To what extent do you prescribe the following pharmacological treatments for sleep maintenance insomnia?

- Ramelteon
- Suvorexant
- Lemborexant
- Eszopiclone
- Zopiclone
- Zolpidem
- Etizolam
- Triazolam
- Flunitrazepam
- Brotizolam
- Nitrazepam
- Trazodone
- Quetiapine
- Traditional Chinese medicine

Q4) To what extent do you implement the following non-pharmacological treatments for sleep maintenance insomnia?

- Sleep hygiene education
- Relaxation therapy
- Stimulus control
- Sleep restriction therapy
- Multi-component cognitive behavioral therapy for insomnia

Q5) To what extent do you prescribe the following pharmacological treatments when insomnia symptoms do not improve with benzodiazepine receptor agonists

- Increase in dosage of benzodiazepine receptor agonists
- Combine current treatment with other benzodiazepine receptor antagonists
- Switching to other benzodiazepine receptor agonists
- Switching to ramelteon
- Switching to suvorexant
- Switching to Lemborexant
- Switching to trazodone
- Switching to quetiapine
- Combine current treatment with ramelteon
- Combine current treatment with suvorexant
- Combine current treatment with Lemborexant
- Combine current treatment with trazodone
- Combine current treatment with quetiapine

Q6) To what extent do you implement the following non-pharmacological treatments when insomnia symptoms do not improve with benzodiazepine receptor agonists

- Differentiate other sleep disorders
- Differentiate comorbid psychiatric disorders
- Refer to a specialist hospital
- Provide sleep hygiene education
- Provide relaxation therapy
- Provide stimulus control
- Provide sleep restriction therapy
- Provide multi-component cognitive behavioral therapy for insomnia

Q7) When do you start benzodiazepine receptor agonists reduction after insomnia symptom improvement?

- Immediately after improvement
- After 1–3 month(s)
- After 3–6 months
- After 6–12 months
- After 12+ months

Q8) To what extent do you use the following strategies when discontinuing benzodiazepine receptor agonists?

- Controlled gradual tapering
- Self- tapering
- Switching to as needed medication
- Switching to another medication
- Provide sleep hygiene education
- Provide relaxation therapy
- Provide stimulus control
- Provide sleep restriction therapy
- Provide multi-component cognitive behavioral therapy for insomnia

Q9) To what extent do you use the following medications when discontinuing benzodiazepine receptor agonists by switching to another medication (substitution methods include additive tapering, incremental and gradual tapering methods, and rapid switching methods)?

- Ramelteon
- Suvorexant
- Lemborexant
- Trazodone
- Quetiapine
- Traditional Chinese medicine

Q10) Which of the following factors are acceptable for patients to continue using benzodiazepine receptor antagonists?

- Patient’s preference to continue benzodiazepine receptor antagonists
- Benzodiazepine receptor antagonists being used as monotherapy or in a low dose
- Lack of awareness of side effects
- Unstable physical or mental states, or low quality of life
- History of exacerbation of insomnia symptoms
- Anticipation of physical and mental deterioration
