# Supplementary figures and images for: Treatment strategies for insomnia in Japanese primary care physicians’ practice: A Web-based questionnaire survey
Source: BMC Prim Care. 2024 Jun 18;25:219. doi: 10.1186/s12875-024-02449-7 (PMC11184713; doi:10.1186/s12875-024-02449-7)

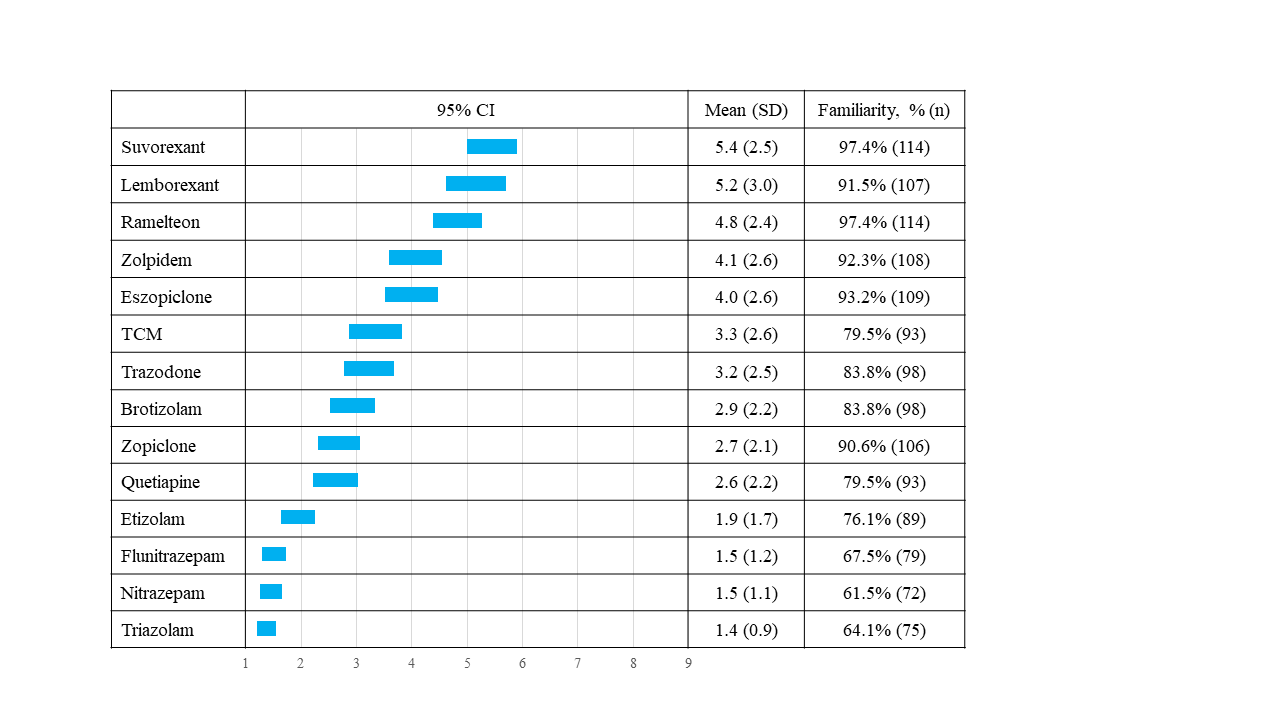

Supplement: Supplementary file 1 — Additional file 1: Figure S1. Pharmacological strategies for sleep onset insomnia. Familiarity indicated the number and percentage of those who knew each pharmacological treatment for sleep onset insomnia. If the participant was familiar with the treatment, they responded to each management option on a nine-point Likert scale (1 = “I do not prescribe it at all”; 9 = “I often prescribe it”). If a participant was not familiar with the option, they were considered to have not prescribed the option at all. Abbreviations: CI, confidence interval; SD, standard deviation; TCM, traditional Chinese medicine. Figure S2. Pharmacological strategies for sleep maintenance insomnia. Familiarity indicated the number and percentage of those who knew each pharmacological treatment for sleep maintenance insomnia. If the participant was familiar with the treatment, they responded to each management option on a nine-point Likert scale (1 = “I do not prescribe it at all”; 9 = “I often prescribe it”). If a participant was not familiar with the option, they were considered to have not prescribed the option at all. Abbreviations: CI, confidence interval; SD, standard deviation; TCM, traditional Chinese medicine. Figure S3. Non-pharmacological strategies for sleep onset insomnia. Familiarity indicated the number and percentage of those who knew each non-pharmacological treatment for sleep onset insomnia. If the participant was familiar with the treatment, they responded to each management option on a nine-point Likert scale (1 = “I do not perform it at all”; 9 = “I often perform it”). If a participant was not familiar with the option, they were considered to have not performed the option at all. Abbreviations: CBT-I, cognitive behavioral therapy for insomnia; CI, confidence interval; SD, standard deviation. Figure S4. Non-pharmacological strategies for sleep maintenance insomnia. Familiarity indicated the number and percentage of those who knew each non-pharmacological treatment for sleep ma [file 12875_2024_2449_MOESM1_ESM.zip › Figure S1.tif]

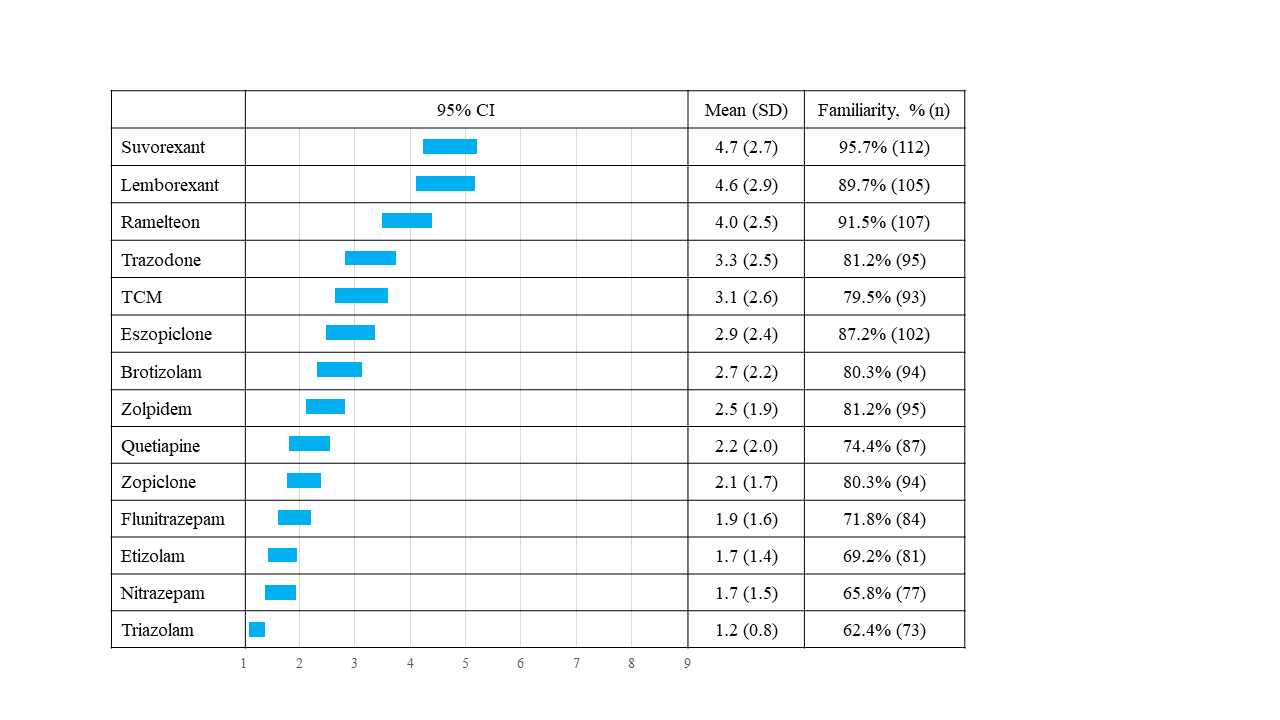

Supplement: Supplementary file 1 — Additional file 1: Figure S1. Pharmacological strategies for sleep onset insomnia. Familiarity indicated the number and percentage of those who knew each pharmacological treatment for sleep onset insomnia. If the participant was familiar with the treatment, they responded to each management option on a nine-point Likert scale (1 = “I do not prescribe it at all”; 9 = “I often prescribe it”). If a participant was not familiar with the option, they were considered to have not prescribed the option at all. Abbreviations: CI, confidence interval; SD, standard deviation; TCM, traditional Chinese medicine. Figure S2. Pharmacological strategies for sleep maintenance insomnia. Familiarity indicated the number and percentage of those who knew each pharmacological treatment for sleep maintenance insomnia. If the participant was familiar with the treatment, they responded to each management option on a nine-point Likert scale (1 = “I do not prescribe it at all”; 9 = “I often prescribe it”). If a participant was not familiar with the option, they were considered to have not prescribed the option at all. Abbreviations: CI, confidence interval; SD, standard deviation; TCM, traditional Chinese medicine. Figure S3. Non-pharmacological strategies for sleep onset insomnia. Familiarity indicated the number and percentage of those who knew each non-pharmacological treatment for sleep onset insomnia. If the participant was familiar with the treatment, they responded to each management option on a nine-point Likert scale (1 = “I do not perform it at all”; 9 = “I often perform it”). If a participant was not familiar with the option, they were considered to have not performed the option at all. Abbreviations: CBT-I, cognitive behavioral therapy for insomnia; CI, confidence interval; SD, standard deviation. Figure S4. Non-pharmacological strategies for sleep maintenance insomnia. Familiarity indicated the number and percentage of those who knew each non-pharmacological treatment for sleep ma [file 12875_2024_2449_MOESM1_ESM.zip › Figure S2.tif]

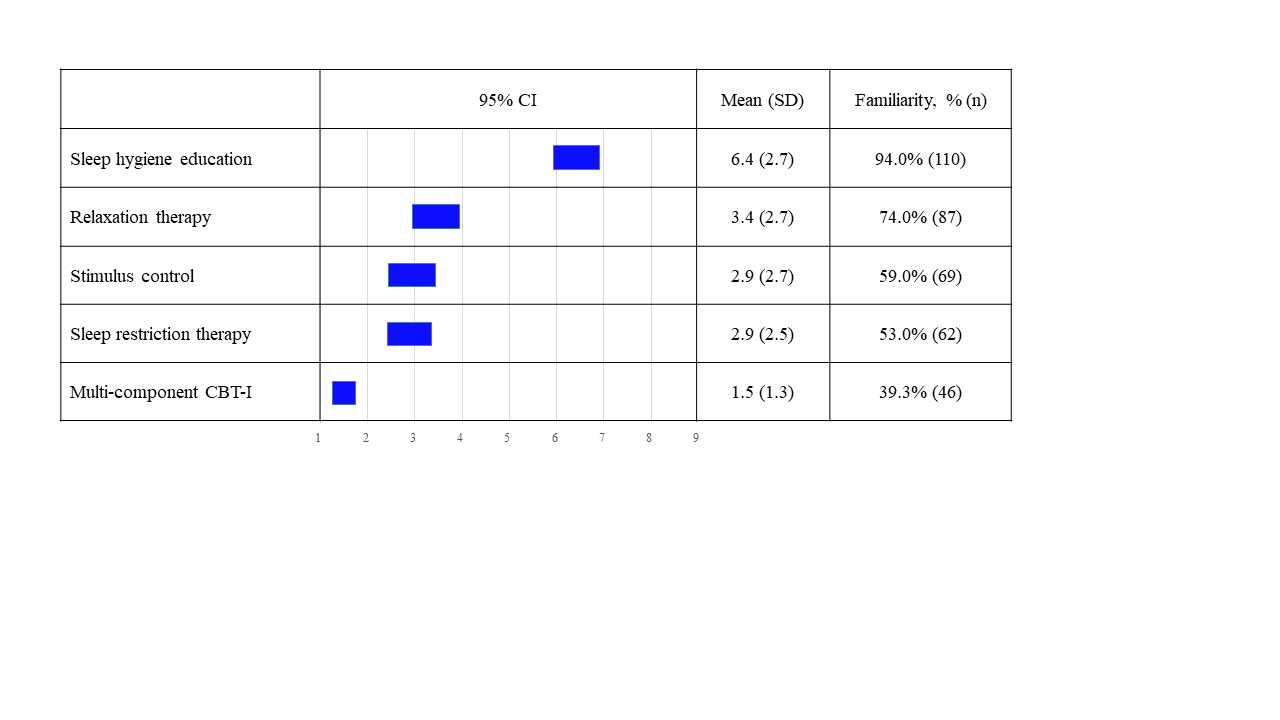

Supplement: Supplementary file 1 — Additional file 1: Figure S1. Pharmacological strategies for sleep onset insomnia. Familiarity indicated the number and percentage of those who knew each pharmacological treatment for sleep onset insomnia. If the participant was familiar with the treatment, they responded to each management option on a nine-point Likert scale (1 = “I do not prescribe it at all”; 9 = “I often prescribe it”). If a participant was not familiar with the option, they were considered to have not prescribed the option at all. Abbreviations: CI, confidence interval; SD, standard deviation; TCM, traditional Chinese medicine. Figure S2. Pharmacological strategies for sleep maintenance insomnia. Familiarity indicated the number and percentage of those who knew each pharmacological treatment for sleep maintenance insomnia. If the participant was familiar with the treatment, they responded to each management option on a nine-point Likert scale (1 = “I do not prescribe it at all”; 9 = “I often prescribe it”). If a participant was not familiar with the option, they were considered to have not prescribed the option at all. Abbreviations: CI, confidence interval; SD, standard deviation; TCM, traditional Chinese medicine. Figure S3. Non-pharmacological strategies for sleep onset insomnia. Familiarity indicated the number and percentage of those who knew each non-pharmacological treatment for sleep onset insomnia. If the participant was familiar with the treatment, they responded to each management option on a nine-point Likert scale (1 = “I do not perform it at all”; 9 = “I often perform it”). If a participant was not familiar with the option, they were considered to have not performed the option at all. Abbreviations: CBT-I, cognitive behavioral therapy for insomnia; CI, confidence interval; SD, standard deviation. Figure S4. Non-pharmacological strategies for sleep maintenance insomnia. Familiarity indicated the number and percentage of those who knew each non-pharmacological treatment for sleep ma [file 12875_2024_2449_MOESM1_ESM.zip › Figure S3.tif]

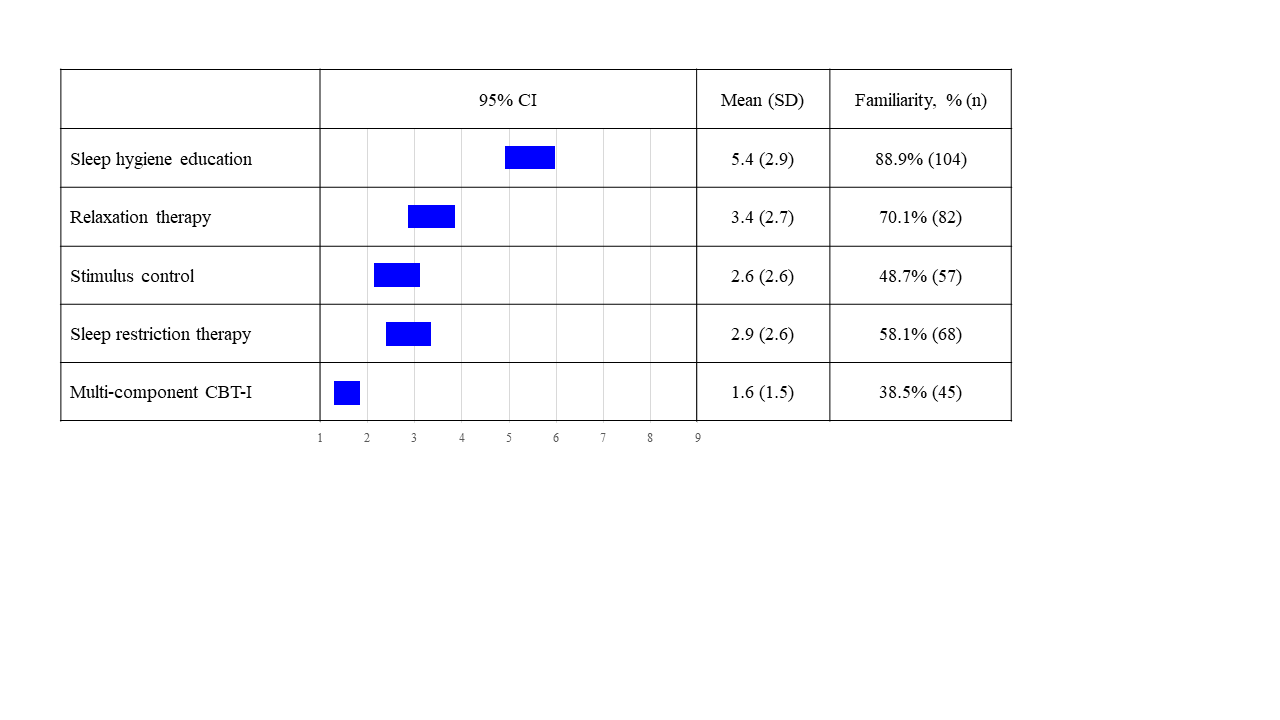

Supplement: Supplementary file 1 — Additional file 1: Figure S1. Pharmacological strategies for sleep onset insomnia. Familiarity indicated the number and percentage of those who knew each pharmacological treatment for sleep onset insomnia. If the participant was familiar with the treatment, they responded to each management option on a nine-point Likert scale (1 = “I do not prescribe it at all”; 9 = “I often prescribe it”). If a participant was not familiar with the option, they were considered to have not prescribed the option at all. Abbreviations: CI, confidence interval; SD, standard deviation; TCM, traditional Chinese medicine. Figure S2. Pharmacological strategies for sleep maintenance insomnia. Familiarity indicated the number and percentage of those who knew each pharmacological treatment for sleep maintenance insomnia. If the participant was familiar with the treatment, they responded to each management option on a nine-point Likert scale (1 = “I do not prescribe it at all”; 9 = “I often prescribe it”). If a participant was not familiar with the option, they were considered to have not prescribed the option at all. Abbreviations: CI, confidence interval; SD, standard deviation; TCM, traditional Chinese medicine. Figure S3. Non-pharmacological strategies for sleep onset insomnia. Familiarity indicated the number and percentage of those who knew each non-pharmacological treatment for sleep onset insomnia. If the participant was familiar with the treatment, they responded to each management option on a nine-point Likert scale (1 = “I do not perform it at all”; 9 = “I often perform it”). If a participant was not familiar with the option, they were considered to have not performed the option at all. Abbreviations: CBT-I, cognitive behavioral therapy for insomnia; CI, confidence interval; SD, standard deviation. Figure S4. Non-pharmacological strategies for sleep maintenance insomnia. Familiarity indicated the number and percentage of those who knew each non-pharmacological treatment for sleep ma [file 12875_2024_2449_MOESM1_ESM.zip › Figure S4.tif]
